# Supplementary material for: Breaking Earth’s shell into a global plate network
Source: Nat Commun. 2020 Jul 17;11:3621. doi: 10.1038/s41467-020-17480-2 (PMC7367830; doi:10.1038/s41467-020-17480-2)
Supplement: Supplementary file 1 — Supplementary Information [file 41467_2020_17480_MOESM1_ESM.pdf]

Nature Communications
Supplementary Information

Breaking Earth's shell into a global plate network

C. A. Tang[1,2], A. A. G. Webb[3]*, W. B. Moore[4,5], Y. Y. Wang[6], T. H. Ma[1,6], T. T. Chen[7]

[1]State Key Laboratory of Coastal & Offshore Engineering, Dalian University of Technology, Dalian 116024, China.
[2]State Key Laboratory of Geological Processes and Mineral Resources, China University of Geosciences (Wuhan) 430074, China.
[3]Department of Earth Sciences and Laboratory for Space Research, University of Hong Kong, Pokfulam Road, Hong Kong 999077, China.
[4]Department of Atmospheric and Planetary Sciences, Hampton University, Hampton, Virginia 23668, USA.
[5]National Institute of Aerospace, Hampton, Virginia 23666, USA.
[6]Deep Underground Research Center, Dalian University of Technology, Dalian 116024, China.
[7]School of Civil and Resources Engineering, Northeastern University, Shenyang 110819, China.

*Correspondence to: aagwebb@hku.hk

Supplementary Figure 1.

Validation of models: Models with a [1] thinner shell with higher elasticity (Model run 8) and a [2] thicker shell with lower elasticity (Model run 9) reveal similar plate breaking results. See “Model Validation” section of Methods and Supplementary Table 1 for further explanation.

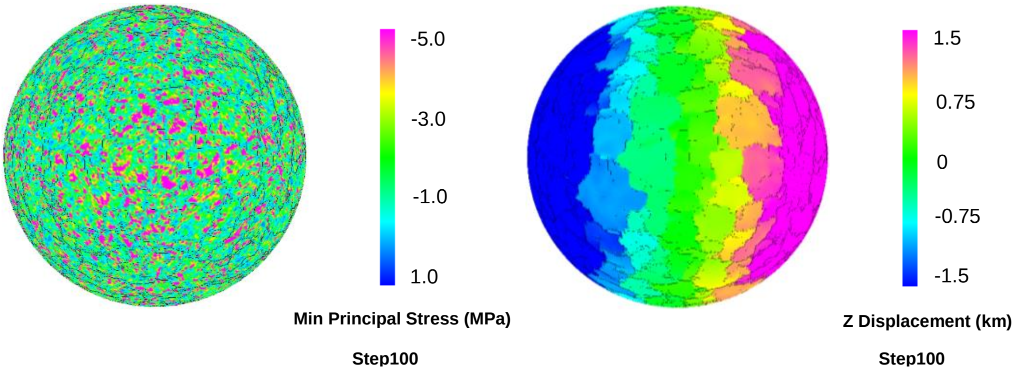

[1] 20km (E=250000MPa, μ =0.04)

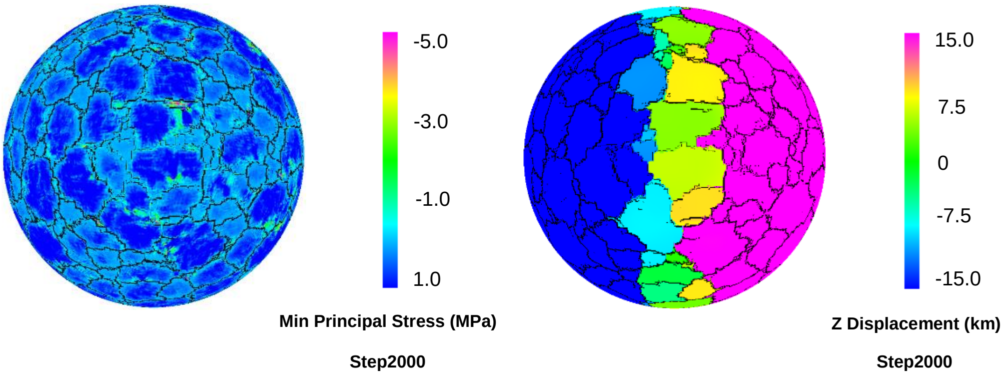

[2] 200km (E=25000MPa, μ =0.4)

Supplementary Figure 2.

Numerically obtained images that demonstrate the complete process of the fracture pattern evolution for Model 1 (parameters listed in Supplementary Table 1). Representations follow after Figure 1, which shows six steps for the reference model (i.e., Model 4). In this figure, fifteen steps are shown for the model run, for both minimum principal stress and displacement. Many of these steps show evolution beyond the thermal expansion limit (see main text and Supplementary Table 1). Similar images are provided for Models 2 and 3 (see Supplementary Figure 3), Models 4 and 5 (see Supplementary Figure 4), Models 6 and 7 (see Supplementary Figure 5), and Models 8 and 9 (see Supplementary Figure 6).

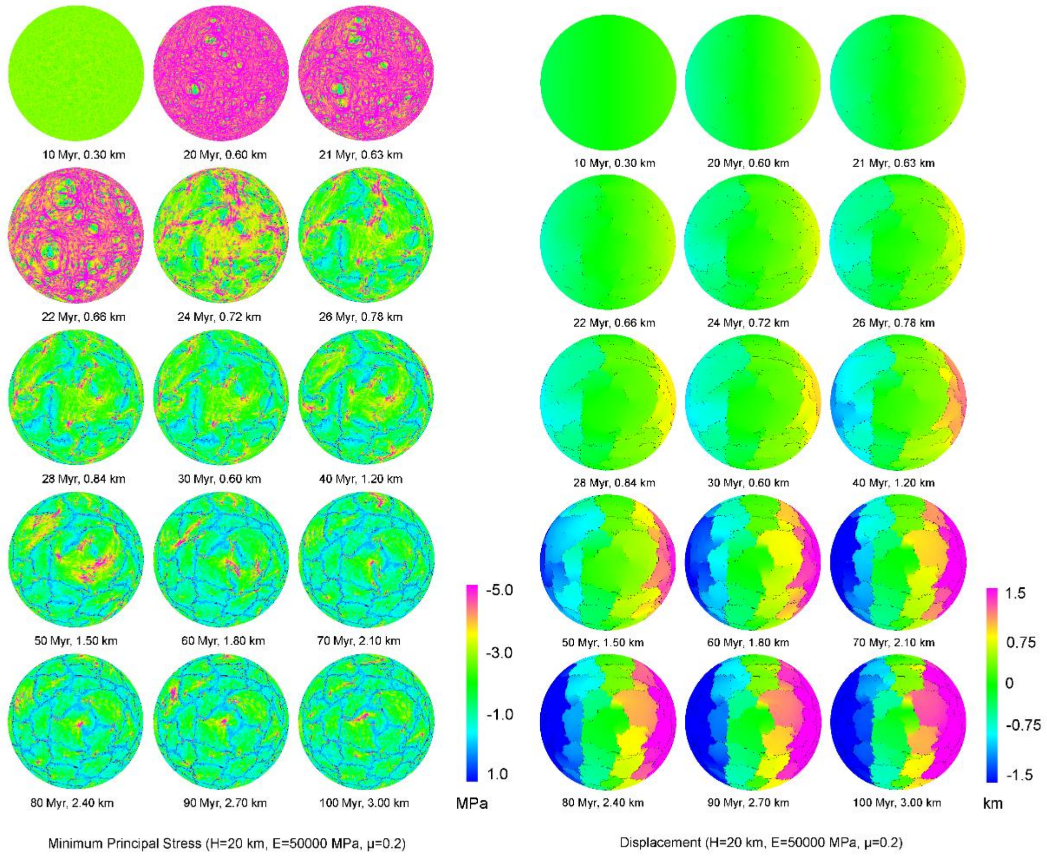

Model 1 (H= 20km, E=50000MPa, μ=0.2)

Supplementary Figure 3.

Numerically obtained images that demonstrate the complete process of the fracture pattern evolution for Models 2 and 3 (parameters listed in Supplementary Table 1). See caption of Supplementary Figure 2 for further details.

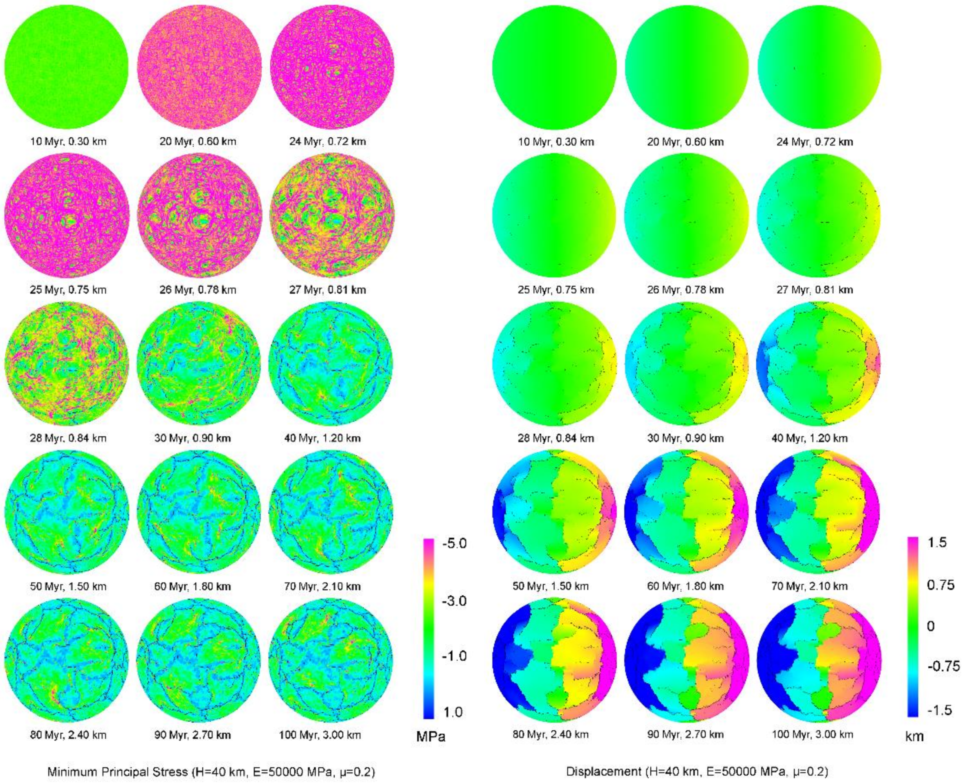

Model 2 (H= 40km, E=50000MPa, μ=0.2)

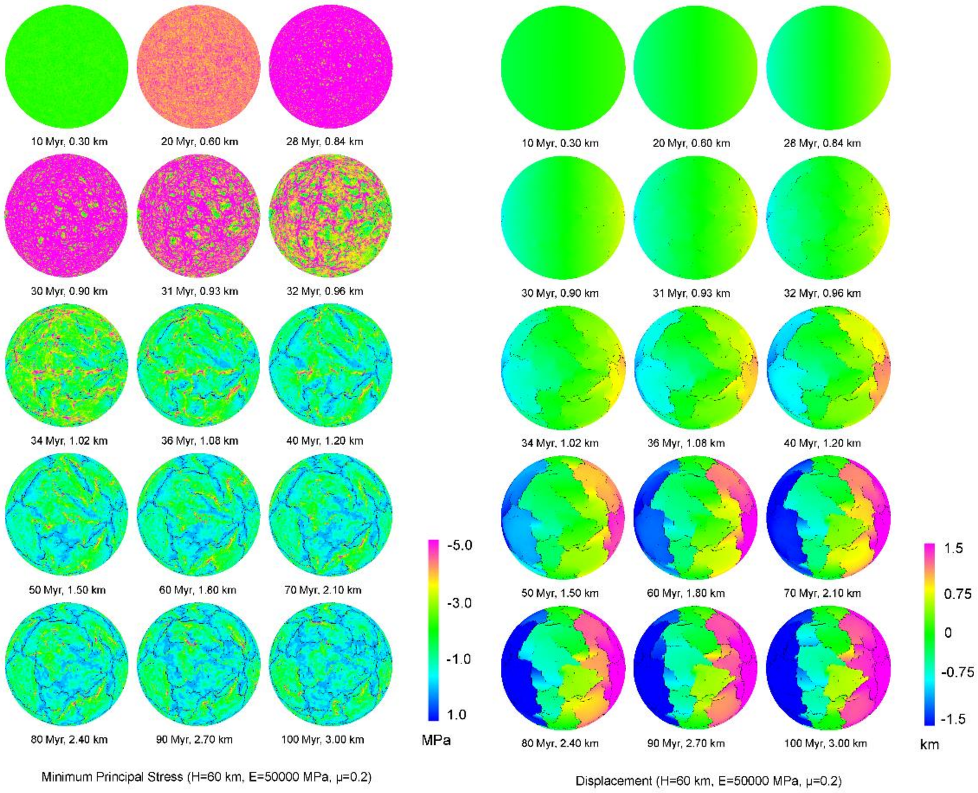

Model 3 (H= 60km, E=50000MPa, μ=0.2)

Supplementary Figure 4.

Numerically obtained images that demonstrate the complete process of the fracture pattern evolution for Models 4 and 5 (parameters listed in Supplementary Table 1). See caption of Supplementary Figure 2 for further details.

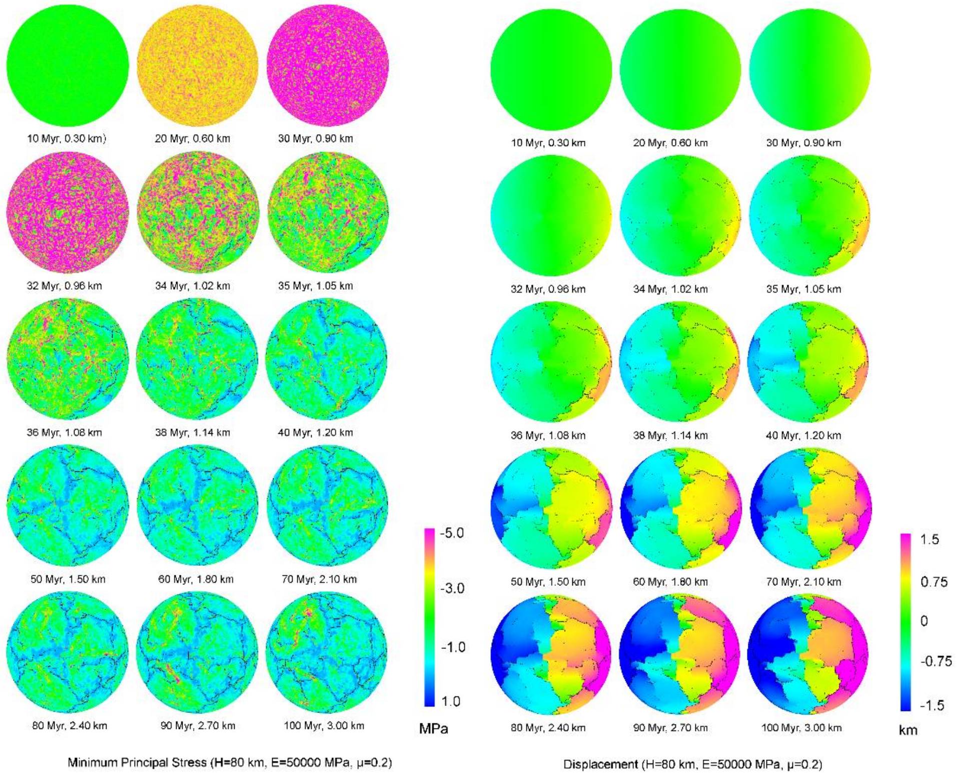

Model 4 (H= 80km, E=50000MPa, μ=0.2)

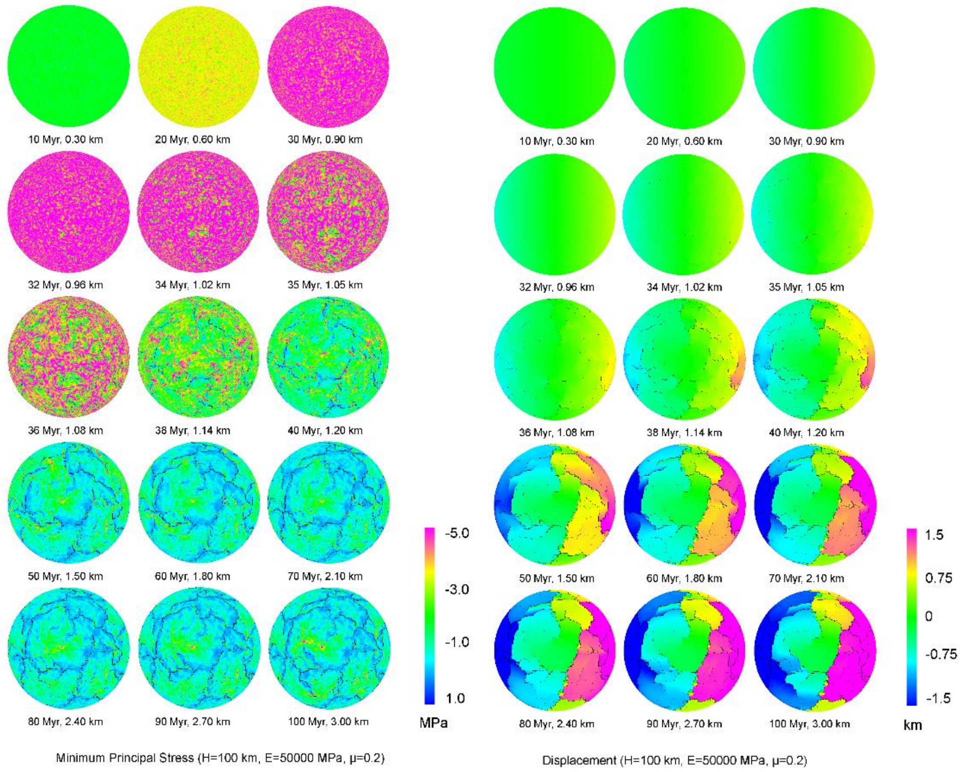

Model 5 (H= 100km, E=50000MPa, μ=0.2)

Supplementary Figure 5.

Numerically obtained images that demonstrate the complete process of the fracture pattern evolution for Models 6 and 7 (parameters listed in Supplementary Table 1). See caption of Supplementary Figure 2 for further details.

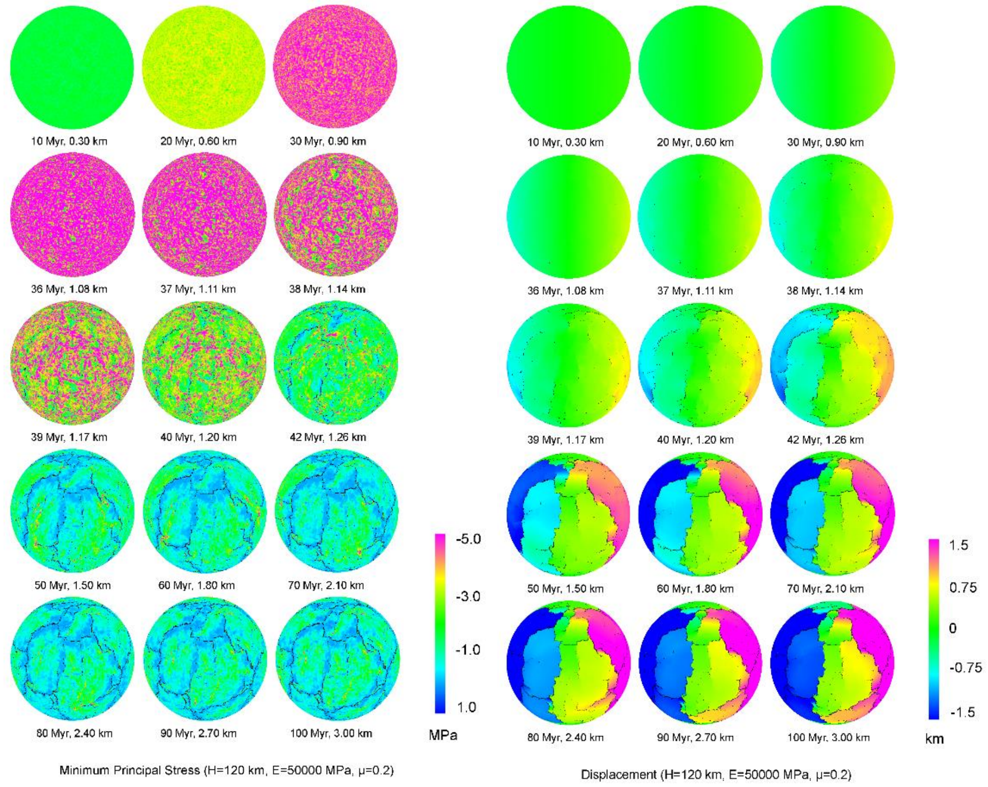

Model 6 (H= 120km, E=50000MPa, μ=0.2)

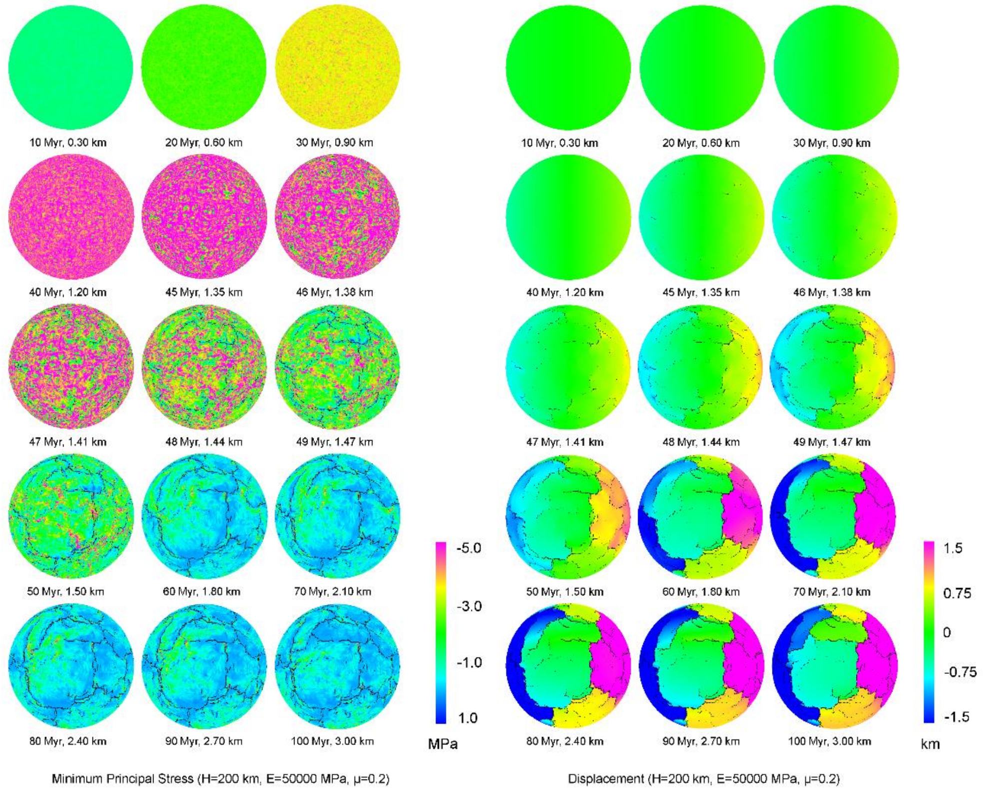

Model 7 (H= 200km, E=50000MPa, μ=0.2)

Supplementary Figure 6.

Numerically obtained images that demonstrate the complete process of the fracture pattern evolution for Models 8 and 9 (parameters listed in Supplementary Table 1). See caption of Supplementary Figure 2 for further details.

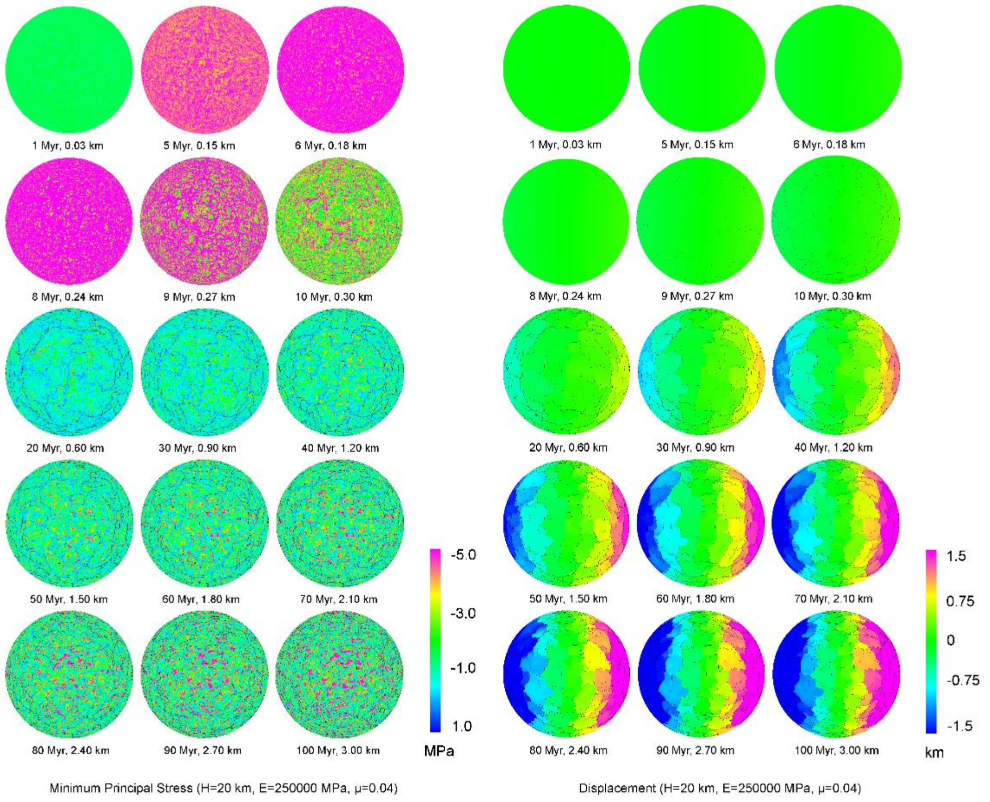

Model 8 (H= 20km, E=250000MPa, μ=0.04)

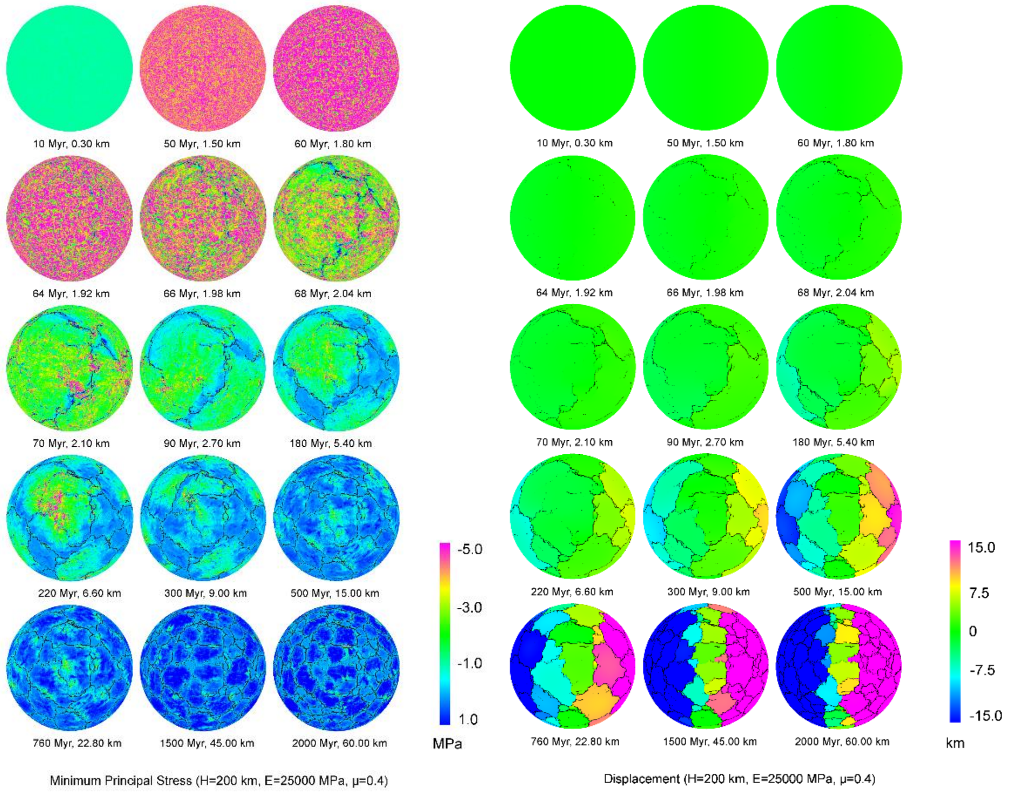

Model 9 (H= 200km, E=25000MPa, μ=0.4)

Supplementary Figure 7.

Fracture growth rate per million year interval (red), and the integrated length of all fractures (blue), as a function of loading steps in time for three model runs (Model 1 – Model 3, see Supplementary Table 1). Dashed lines indicate the evolution beyond the thermal expansion limit (see main text).

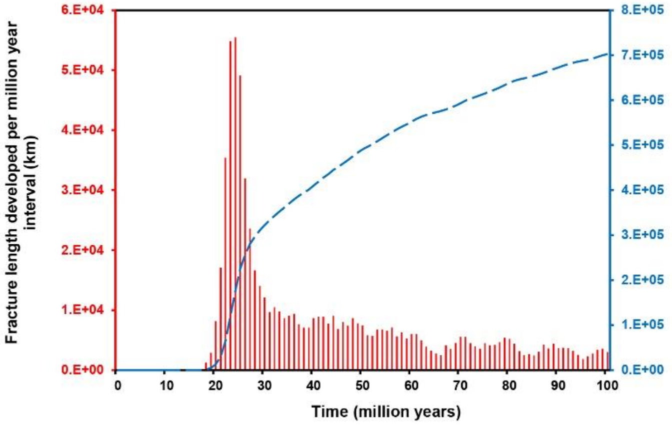

Model 1 (H= 20km, E=50000MPa, μ=0.2)

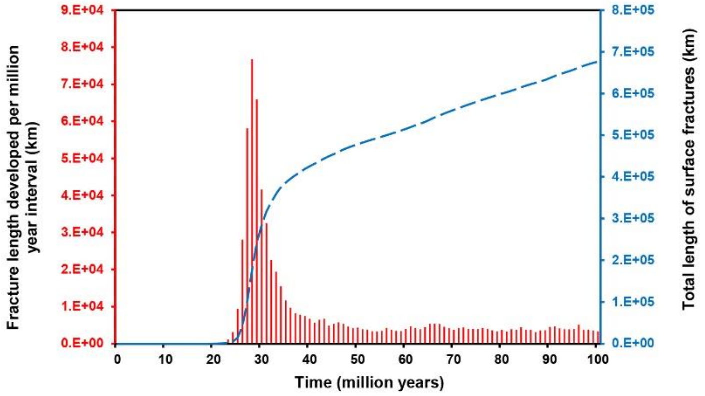

Model 2 (H= 40km, E=50000MPa, μ =0.2)

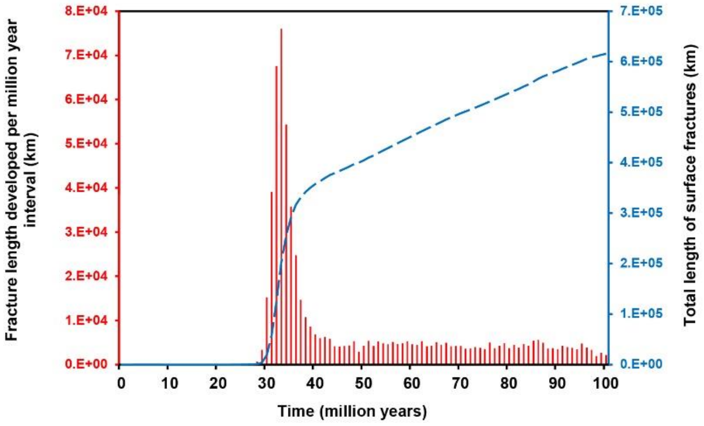

Model 3 (H= 60km, E=50000MPa, μ =0.2)

Supplementary Figure 8.

Fracture growth rate per million year interval (red), and the integrated length of all fractures (blue), as a function of loading steps in time for three model runs (Model 4 – Model 6, see Supplementary Table 1). Dashed lines indicate the evolution beyond the thermal expansion limit (see main text).

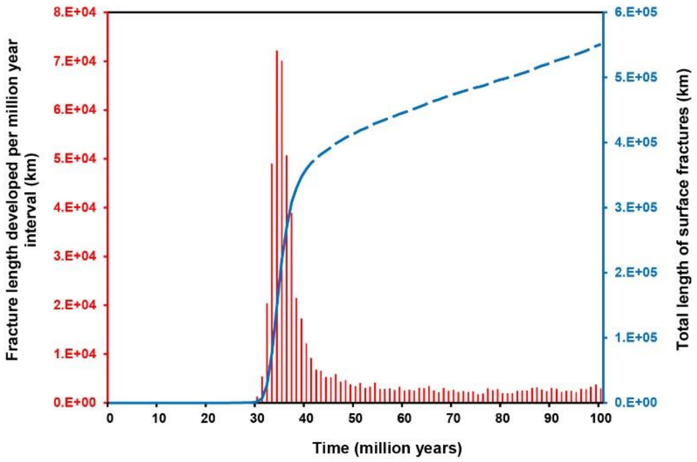

Model 4 (H= 80km, E=50000MPa, μ =0.2)

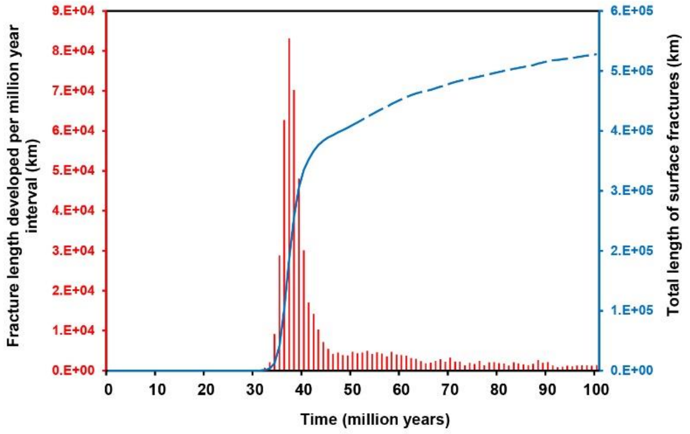

Model 5 (H= 100km, E=50000MPa, μ =0.2)

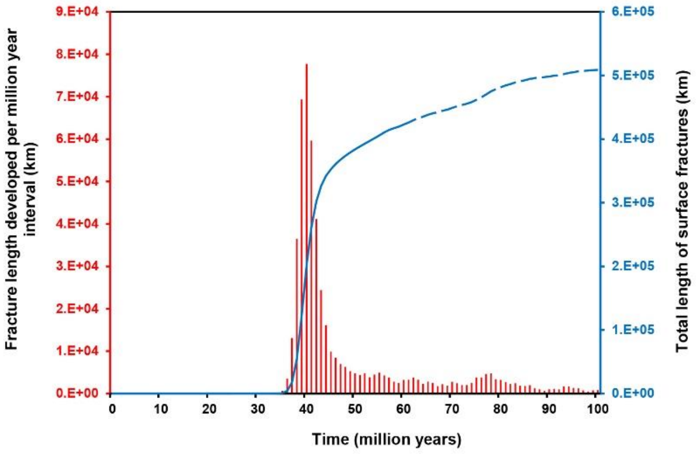

Model 6 (H= 120km, E=50000MPa, μ =0.2)

Supplementary Figure 9.

Fracture growth rate per million year interval (red), and the integrated length of all fractures (blue), as a function of loading steps in time for three model runs (Model 7 – Model 9, see Supplementary Table 1). Dashed lines indicate the evolution beyond the thermal expansion limit (see main text).

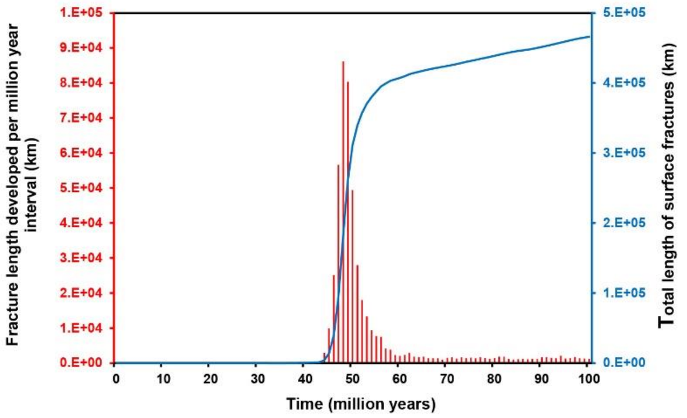

Model 7 (H= 200km, E=50000MPa, μ =0.2)

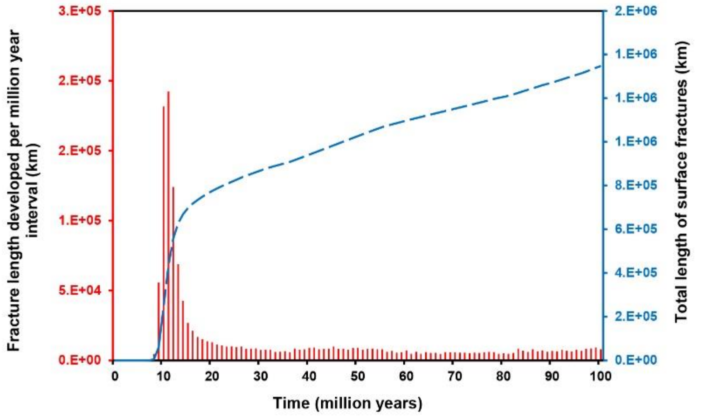

Model 8 (H= 20km, E=250000MPa, μ =0.04)

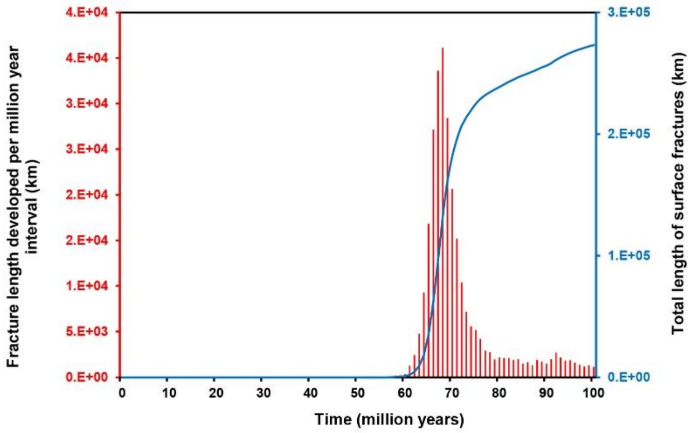

Model 9 (H= 200km, E=25000MPa, μ =0.4)

Supplementary Figure 10.

This plot illustrates how model runs with thicker shells produce less fracture length and fewer plates over the same period (at step 100, i.e., after 100 million years in the models).

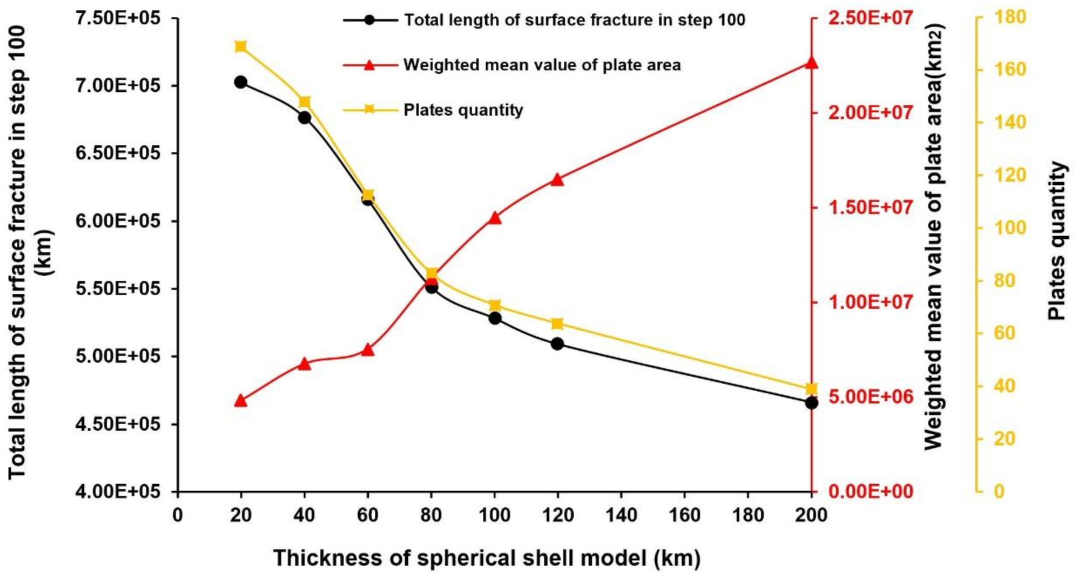

Supplementary Figure 11.

The two plots above show the integrated length of surface fractures against loading parameters for three model runs. Dashed lines indicate the evolution beyond the thermal expansion limit (see main text). In both plots, the 80 km reference model undergoes radial expansion at three different rates: 0.015 km/Myr, 0.03 km/Myr (the rate in all other models in this work), and 0.06 km/Myr, equivalent to radial loading of 15 m, 30 m, and 60 m per time step. The upper plot shows the results in terms of radial loading value (on the x-axis), demonstrating that for all rates a phase of rapid fracture growth initiates shortly before a radial loading of 1 km is attained. The lower plot shows these same fracture growth results in terms of model time (on the x-axis), displaying how the slower the loading rate, the longer it takes to initiate rapid fracture growth. The main overall features seen in all of our models – an early period of rapid fracture growth followed by a slower period of fracture ingrowth after the fracture network is largely established – persist for all three different loading rates.

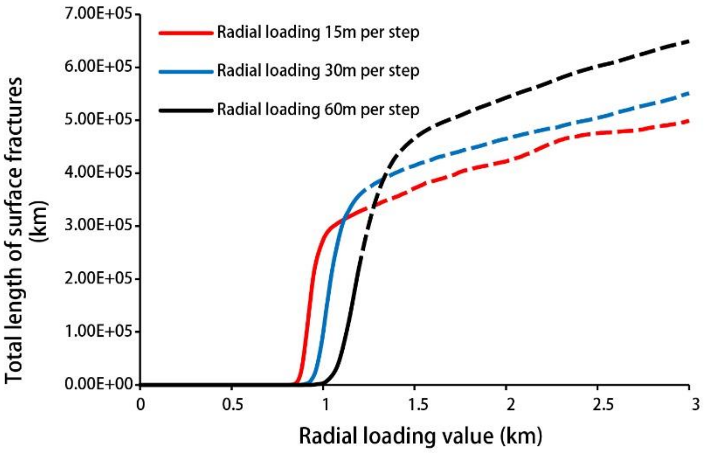

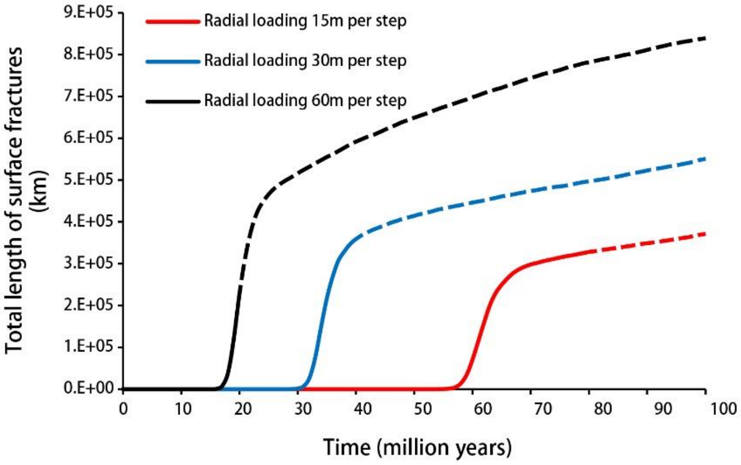

Supplementary Figure 12.

Finite element method model with a heterogeneous spherical shell loaded from inside. The model consists of 1 million elements. We load the whole shell from the interior boundary, and the top boundary is free to displace as necessary. We use a constant displacement increment along the interior boundary.

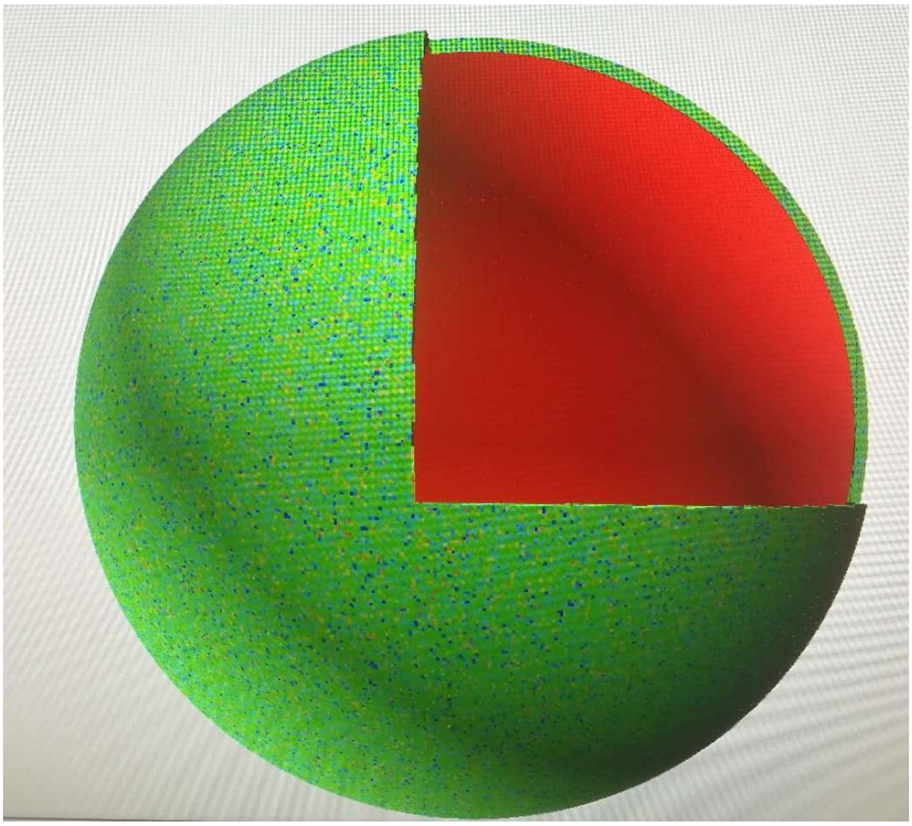

Supplementary Figure 13.
Constitutive law for uniaxial compressive and tensile stress.

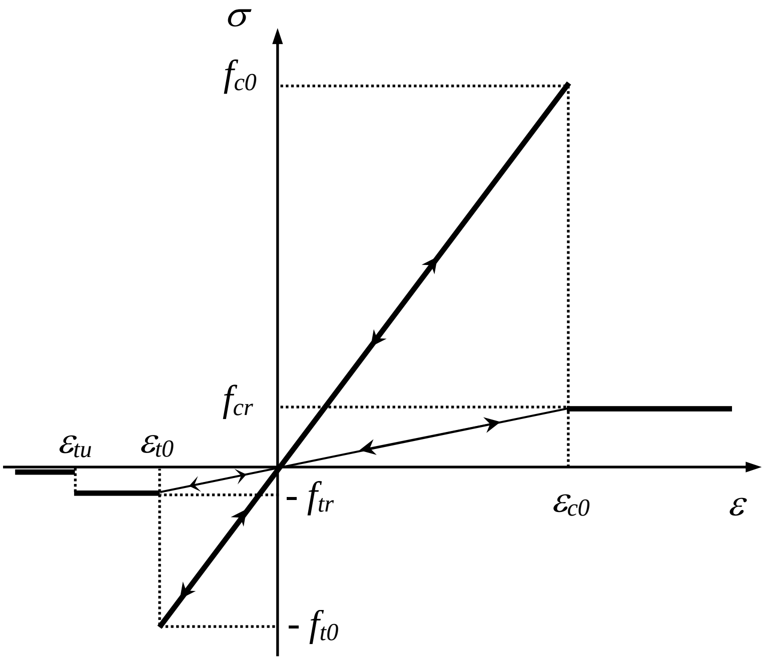

Supplementary Table 1.

Mechanical and geometric parameters for the spherical shell simulation models (Models 1 through 9), with the maximum uplift from thermal expansion listed in the right column. Model 4 is the reference model (see main text). The maximum thermal expansion is given by (α)*(dT)*(D), where α is ~3*10^5 per °C, dT is the average temperature increase (500 °C), and D is lithospheric thickness.

	Shell thickness (km)	Elastic module (MPa)	Poisson's ratio	Compressive strength (MPa)	Tensile strength (MPa)	Inhomogeneity Index	Thermal Expansion Uplift Limit (km)
Model 1	20	50,000	0.2	100	10	4	0.3
Model 2	40	50,000	0.2	100	10	4	0.6
Model 3	60	50,000	0.2	100	10	4	0.9
Model 4	80	50,000	0.2	100	10	4	1.2
Model 5	100	50,000	0.2	100	10	4	1.5
Model 6	120	50,000	0.2	100	10	4	1.8
Model 7	200	50,000	0.2	100	10	4	3
Model 8	20	250,000	0.04	100	10	4	0.3
Model 9	200	25,000	0.4	100	10	4	3
