# Supplementary material for: Breaking Earth’s shell into a global plate network
Source: Nat Commun. 2020 Jul 17;11:3621. doi: 10.1038/s41467-020-17480-2 (PMC7367830; doi:10.1038/s41467-020-17480-2)
Supplement: Supplementary file 3 — Description of Additional Supplementary Files [file 41467_2020_17480_MOESM3_ESM.pdf]

Description of Additional Supplementary Files

File Name: Supplementary Movie 1
Description: As in Figure 1, this movie shows the reference model with an 80 km thick lithosphere showing the progressive development of a global fracture network in response to displacement-controlled increasing interior pressure (at 0.03 km per million years (m.y.)). Specifically, the sphere shows the evolution of the global surface distribution of minimum principal stress magnitudes through 100 million years of model time (each "step" represents 1 m.y. of model time). As discussed in the main text, by ~40 m.y. a global fracture network is established, dividing the surface of the sphere into platelike segments. 40 m.y. also represents the thermal expansion limit (see main text).

File Name: Supplementary Movie 2
Description: This shows the same evolution as Supplementary Movie 1, but in a rotating reference frame.

File Name: Supplementary Movie 3
Description: This shows the same evolution as Supplementary Movie 1, but showing the surface displacement (rather that the stress), articulated as in Figure 1.
