# Supplementary figures and images for: Breaking Earth’s shell into a global plate network
Source: Nat Commun. 2020 Jul 17;11:3621. doi: 10.1038/s41467-020-17480-2 (PMC7367830; doi:10.1038/s41467-020-17480-2)

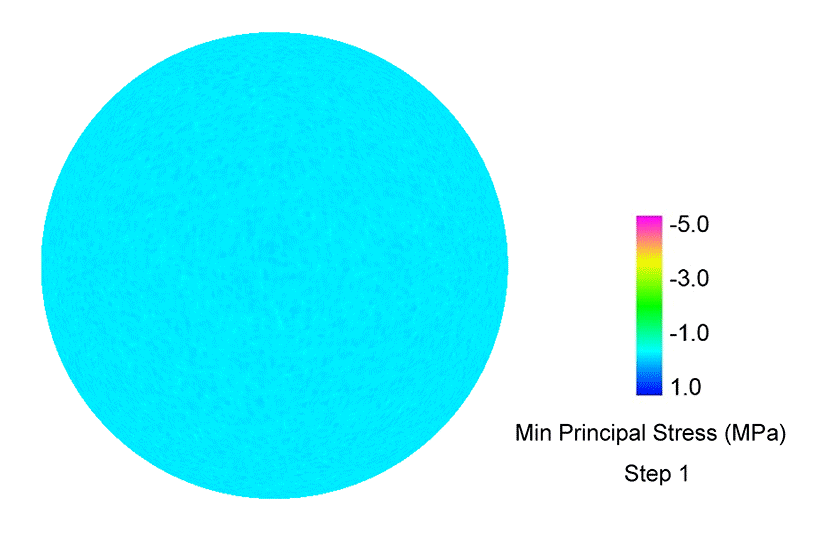

Supplement: Supplementary file 4 — Supplementary Movie 1 [file 41467_2020_17480_MOESM4_ESM.gif]

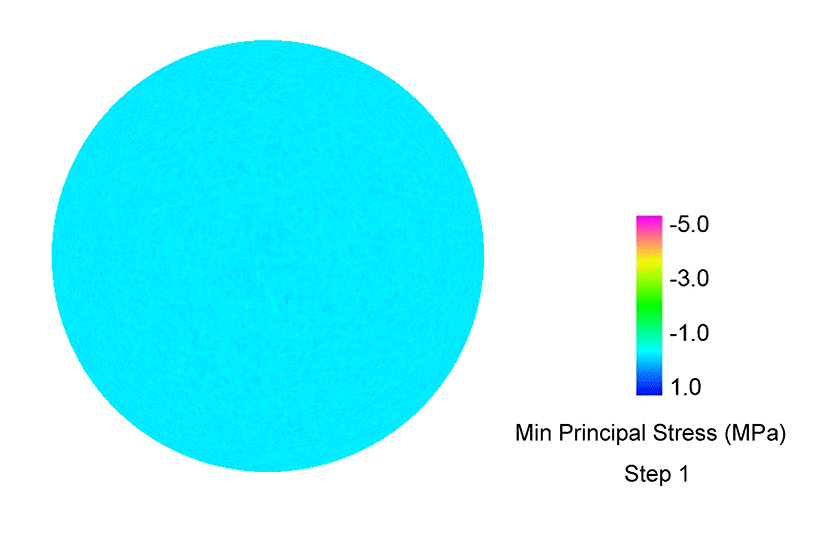

Supplement: Supplementary file 5 — Supplementary Movie 2 [file 41467_2020_17480_MOESM5_ESM.gif]

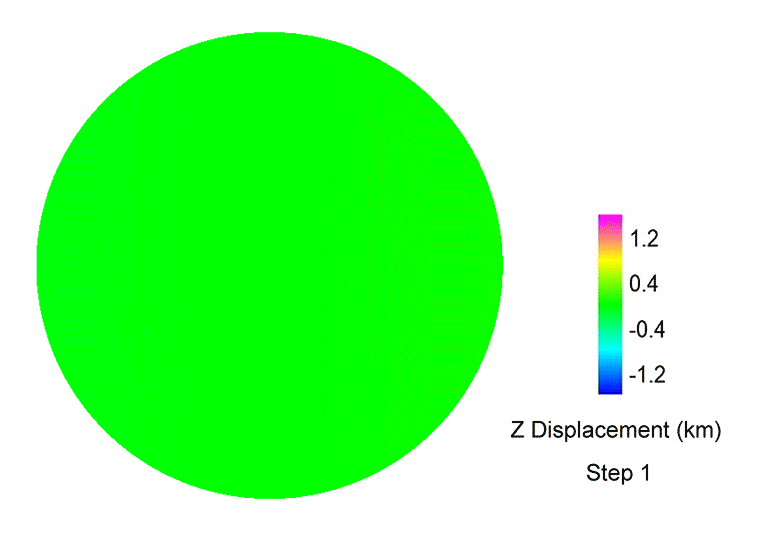

Supplement: Supplementary file 6 — Supplementary Movie 3 [file 41467_2020_17480_MOESM6_ESM.gif]
